# Fetal Window of Vulnerability to Airborne Polycyclic Aromatic Hydrocarbons on Proportional Intrauterine Growth Restriction

**DOI:** 10.1371/journal.pone.0035464

**Published:** 2012-04-24

**Authors:** Hyunok Choi, Lu Wang, Xihong Lin, John D. Spengler, Frederica P. Perera

**Affiliations:** 1 Department of Environmental Health, Harvard School of Public Health, Boston, Massachusetts, United States of America; 2 Department of Biostatistics, The University of Michigan, Ann Arbor, Michigan, United States of America; 3 Department of Biostatistics, Harvard School of Public Health, Boston, Massachusetts, United States of America; 4 Columbia Center for Children's Environmental Health, Mailman School of Public Health, New York, New York, United States of America; National University of Singapore, Singapore

## Abstract

**Background:**

Although the entire duration of fetal development is generally considered a highly susceptible period, it is of public health interest to determine a narrower window of heightened vulnerability to polycyclic aromatic hydrocarbons (PAHs) in humans. We posited that exposure to PAHs during the first trimester impairs fetal growth more severely than a similar level of exposure during the subsequent trimesters.

**Methods:**

In a group of healthy, non-smoking pregnant women with no known risks of adverse birth outcomes, personal exposure to eight airborne PAHs was monitored once during the second trimester for the entire cohort (n = 344), and once each trimester within a subset (n = 77). Both air monitoring and self-reported PAH exposure data were used in order to statistically estimate PAH exposure during the entire gestational period for each individual newborn.

**Results:**

One natural-log unit increase in prenatal exposure to the eight summed PAHs during the first trimester was associated with the largest decrement in the Fetal Growth Ratio (FGR) (−3%, 95% Confidence Interval (CI), −5 to −0%), birthweight (−105 g, 95% CI, −188 to −22 g), and birth length (−0.78 cm, 95% CI, −1.30 to −0.26 cm), compared to the unit effects of PAHs during the subsequent trimesters, after accounting for confounders. Furthermore, a unit exposure during the first trimester was associated with the largest elevation in Cephalization Index (head to weight ratio) (3 μm/g, 95% CI, 1 to 5 μm/g). PAH exposure was not associated with evidence of asymmetric growth restriction in this cohort.

**Conclusion:**

PAH exposure appears to exert the greatest adverse effect on fetal growth during the first trimester. The present data support the need for the protection of pregnant women and the embryo/fetus, particularly during the earliest stage of pregnancy.

## Introduction

Polycyclic aromatic hydrocarbons (PAHs) are multiphasic fused aromatic rings of carbon compounds. Ubiquitous human dependence on combustion of carbon-containing materials—primarily fossil fuel—has contributed to PAH air pollution as a global issue [1], [2], [3]. Some PAHs are potent mutagens, genotoxins and known human carcinogens [4].

Transplacental mutagenicity and genotoxicity of some PAHs are well established in several experimental animal species [5], [6]. For example, *in utero* exposure of mice to benzo[*a*]pyrene (B[*a*]P) and dibenzo[*a,l*]pyrene could induce the formation of DNA-adducts in thymocytes and splenocytes of the offspring [7]. Such adducts are vital precursors to PAH-mediated carcinogenesis [7]. *In utero* PAH exposure could also induce lung and liver tumors, as well as lymphoma in mice offspring [8], [9], [10].

In humans, maternal exposure to certain carcinogenic PAHs (c-PAHs) during pregnancy could induce DNA damage, histone modification, and chromosome abnormalities in the fetus [11] at an environmentally relevant exposure range. PAH-DNA adducts have been detected in human fetal umbilical cord blood DNA, as well as in maternal blood after exposure to ambient airborne PAHs [12]. Prenatal PAH exposure quantified using personal air monitoring significantly predicted dose-responsive elevation in chromosomal aberrations in cord blood [13]. Accordingly, prenatal PAH exposure may increase cancer risk in humans.

Furthermore, prenatal exposure to PAHs through maternal inhalation is associated with a wide range of non-carcinogenic fetotoxic effects, including intrauterine growth restriction [14], small-for-gestational age [15], and preterm delivery [15]. When the prenatally monitored group of newborns was followed to school age, the prenatal PAH exposure furthermore impaired neurodevelopmental performances [4], and increased the likelihood of asthma-related symptoms [11].

However, to date, the precise window of fetal susceptibility to the airborne PAHs on adverse birth outcomes has never been directly examined in human populations. The importance of this question stems from the observed variability in transplacental fetotoxic effects according to the gestational window of exposure in several animal models [8], [16], [17]. Thus, not only the concentration and the components of the PAH mixture in air, but also the fetal age, might influence the type and the severity of adverse clinical health outcomes in humans.

The goal of the present analysis is to address this critical gap in knowledge. Several lines of experimental and clinical evidence suggest that the embryo/fetus is most vulnerable to a number of PAHs during the first trimester, or the period of organogenesis. Two modes of PAH effects have been identified during the earliest gestational weeks, the first being the interference with placental development [18], [19], [20], and the second being direct injury of the embryo. In the first mechanism, PAHs, in particular B[*a*]P, disrupt early trophoblast endovascular proliferation and their ability to infiltrate into the fetal envelope [21]. Such proliferative failure results in an altered vascular labyrinthine structure of the placenta, a lowered fetoplacental vascular surface area, and altered apoptosis in fetal endothelia and syncytiotrophoblast cells [10], [22]. This critically impairs the development of several vital fetal organ systems, including the central nervous systems and the heart, as they undergo terminal cell lineage commitment during this period [10], [22]. In the second mode of PAH fetotoxicity, the direct impairment of embryonic growth differs with the gestational age of exposure as well as the target organs [17]. For example, B[*a*]P administration in the patas monkey model has shown that the fetal brain is most vulnerable to B[*a*]P DNA adduct formation during the first trimester, while the fetal liver is most vulnerable to the same adducts during the second trimester [17].

A small body of epidemiological evidence suggests that ambient PAH concentrations pose the greatest risk during the first trimester. In Teplice, Czech Republic, an area noted for high environmental pollution, exposure of pregnant women to high ambient concentration of c-PAHs and PM_10_ during the first gestational month was associated with significantly increased risk of intrauterine growth restriction [14], [23].

Considering both biological and epidemiological evidence, we tested the hypothesis that an embryo/fetus is most vulnerable during the first trimester per unit PAH exposure, compared to a comparable unit of exposure during the second or the third trimester in a cohort of non-smoking, healthy pregnant women. We examined this by 1) using a newly developed model to estimate personal exposure to airborne PAHs during the entire gestational period for each newborn; 2) comparing the birth outcome effect sizes per unit PAH exposure during given window of interest (i.e. trimester or gestational month) to those during other periods, including the sixth gestational month as the reference period; 3) examining whether the gestational period of IUGR onset is associated with an IUGR subtype as well as its severity.

## Methods

### Site Characterization

In a prospective birth cohort study, pregnant women were recruited from prenatal care clinics during their first trimester in Krakow, Poland. In the city of Krakow, coal combustion for domestic heating represents the major air pollution source [24]. In contrast, automobile traffic emissions and coal-combustion for industrial activities are relatively minor contributors [24]. During typical winter days in 2005, ambient PM_10_ concentrations in Krakow have been shown to peak at 400 μg/m^3^ [24]. During such episodes, ambient B[*a*]P and other PAHs were spatially homogeneous in their concentrations over the city, suggesting that the city's population could have been exposed to a narrow range of concentrations [24].

### Study Subjects

Details regarding subject enrollment and methods are discussed elsewhere [25]. We targeted Caucasian pregnant women of ethnic Polish background during the 8^th^ to 13^th^ weeks of gestation. To reduce confounding, only young (age, 18–35) and healthy women with no known risks for adverse birth outcomes were eligible. Those who met all the eligibility criteria were simultaneously monitored for their personal (n = 344), home indoor (n = 76), and outdoor (n = 70) levels of PAHs and PM_2.5_ during the second trimester of pregnancy between November 2000 and January 2003 [25]. The women also answered a questionnaire on health, lifestyle and exposure history. In the subset of women (n = 77), they were monitored for their personal exposure, in addition to the indoor and outdoor monitoring of PAHs. In the subset, personal monitoring was repeatedly taken once during each trimester (see Table S1). For personal monitoring, each woman carried her a personal air monitor which operated for a consecutive 48-hour period. The split flow inlet, placed near the woman's breathing zone, drew in the particulate or semi-volatile vapor PAHs and particles ≤2.5 μm (PM_2.5_) on a pre-cleaned quartz microfiber filter and polyurethane foam backup. The filters were analyzed for pyrene and eight PAHs known to be carcinogenic as well as having other toxicities: benz(*a*)anthracene, chrysene/isochrysene, benzo(*b*)fluoranthene, benzo(*k*)fluoranthene, benzo(*a*)pyrene, indeno(*1,2,3-cd*)pyrene, dibenz(*a,h*)anthracene and benzo(*g,h,i*)perylene.

Full enrollment criteria required the completion of a prenatal interview, compliance with air monitoring with minimal problems in data quality, and the donation of a maternal and/or newborn cord blood sample at delivery. The cotinine concentration (0.319±0.882 ng/ml serum, mean ± S.D.), which was available for 228 newborns, indicated no secondhand smoke exposure (≥15 ng/mL) in women up to 48-hours prior to delivery. The study was reviewed and approved by the institutional review boards of Jagiellonian University in Krakow and Columbia Presbyterian Medical Center, New York City, US. Written informed consent was obtained from all study participants.

### Outcomes

In addition to abstracting birthweight (g), birth length (cm) and birth head circumference (cm) from the medical record, we calculated fetal growth ratio (FGR, %), Cephalization Index (cm/g), and Ponderal Index (g/cm^3^) for each newborn. In order to test our main hypothesis, we chose fetal growth restriction (FGR) as a marker for symmetric IUGR because of its clinical predictiveness of neonatal morbidities [26], advantage in linear regression analysis, and its consistency with small for gestational age (SGA) outcome in our New York City birth cohort [15]. FGR indicates percent underweight relative to the population mean [26]. Moderate to severe (i.e. <80%) FGR has been associated with a greater risk of delayed neurodevelopment [27], [28], shorter stature, cardiovascular disease, insulin resistance and diabetes during adulthood [29]. In order to meet our third specific aim of detecting severe IUGR subtypes, we chose two indices of asymmetric fetal growth, the Cephalization Index and the Ponderal Index. The Cephalization Index has been validated as a marker for a severe IUGR subtype for an impaired fetal brain development [26]. Ten-year old children born with larger head size relative to their body weight (i.e. “brain spared”) scored significantly lower in neurodevelopmental tests, intelligence quotient, and school performance [30]. In a murine model, fetal cranium and neural tissues are most exquisitely sensitive to PAHs during the period of organogenesis [31]. Accordingly, we speculated that onset of PAH-mediated impairment in fetal neural tissues occurs in late first to early second trimester. On the other hand, the Ponderal Index was chosen because earlier epidemiologic studies observed that the SGA newborns who were particularly “thin” were at a greater risk of perinatal asphyxia and extended hospitalization, compared with the proportional SGA cases [32]. Low Ponderal Index furthermore predicted shorter stature, cardiovascular disease, insulin resistance, and diabetes during adulthood [29]. As fetal weight gain is highest during the third trimester, such impairment is expected to result in low Ponderal Index [33]. Accordingly, we posited that the Ponderal Index captures an acute IUGR phenomenon with the third trimester as the time point of onset. The Ponderal Index was calculated as [birthweight (g)/(birthlength)^3^ (cm)^3^×100] [32]. The Cephalization Index was calculated as the ratio of the [birth head circumference (μm)/birthweight (g)] [30]. FGR was calculated as observed birthweight/mean birthweight at a given gestational age and gender based on 1994–1996 Polish birthweight distribution [34]. Cephalization index has been validated as a marker for a severe IUGR subtype [26].

### Statistical Analysis

As in prior analyses, the main exposure variable was calculated as a summed concentration of the eight carcinogenic PAHs (Σ8 c-PAHs) [35]. The exposure unit of interest was a one natural-log (ln) unit increase in concentration of Σ8 c-PAHs.

#### Personal Exposure Concentration Prediction

We considered 48-hour personal exposure monitored data in the overall cohort and the subset (see Table S1) in order to predict chronic prenatal exposure of the same newborns during the unmonitored periods. Each individual newborn's exposure to Σ8 c-PAHs over the entire gestational period was estimated using two approaches – a random effects model and a semi-parametric mixed effects model.

First, based on our earlier analysis [25], the individual newborn's chronic prenatal exposure was estimated using a random effects model as shown below in [1].
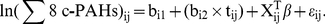
(1)Personal exposure to 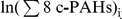 for the 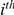 subject at the 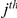 measurement was modeled as a function of the random intercept (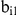), the product of random slope (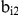) and calender time 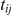, and a vector of other covariates, which are time-independent (e.g., living in City center, secondhand smoke exposure), denoted as 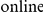. The measurement error is noted as 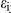. The superscript ^T^ represents the vector of each covariate, and *β* the associated regression coefficient. Here, 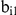, 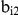 and 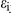 are expected to be normally distributed with a mean of 0. By including both random intercept and random slope, the predicted individual PAH exposure trajectory not only shift from the population mean curve by a subject specific amount, b_i1_, but also has a subject specific slope, 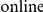. To reduce within-person collinearity in predicted personal exposure, mean over three months period of personal exposure to 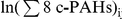 was taken for each person during each trimester.

Alternatively, we estimated individual's chronic gestational exposure using a semi-parametric mixed model [25] as shown in [2]. 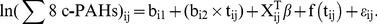
(2)Personal exposure to 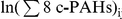 for the 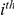 subject at the 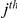 measurement was modeled as a function of the random intercept (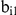), the product of random slope (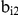) and calender time 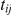, and a vector of other time-independent covariates (i.e., living in city center, secondhand smoke, parity, maternal pre-pregnancy body mass index, gestational age, newborn gender, and c-section delivery for birth head circumference and Cephalization Index only) (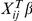), where *^T^* stands for a transpose of a vector of the covariates, and *β* denotes the corresponding coefficients. The nonlinear trend in ambient PAH levels was captured by a fully nonparametric function, f(t_ij_). Term, e_ij_, denotes a measurement error. The distributions of 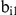, 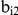 and 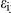 were assumed to be normal with a mean of 0. Overall, the semi-parametric model yielded more precise predicted concentrations of personal exposure than the estimates using the linear mixed effects model during each gestational month (see Figure S1). Considering the observed cohort's mean personal exposure throughout the monitoring period as the gold—standard, Pearson's coefficients between predicted and observed personal exposure levels were 0.91, 0.98 and 0.96 for the third, sixth and the eighth gestational month using semi-parametric mixed model (Figure S1). In comparison, the linear mixed effects model, Pearson's coefficient between predicted and observed concentration was 0.76 during the sixth gestational month.

#### Parametric Analysis of the Birth Outcomes

Functional linear models of the i^th^ subject's birth outcome is shown as, 
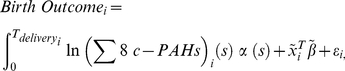
(3)where the fetal growth is a function of the integrated chronic exposure between conception and delivery, 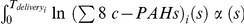, and other time-independent, potential confounders (i.e., gestational age, newborn gender, parity, and the mother's pre-pregnancy body mass index) 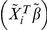, and measurement error (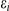) [36]. Here, 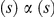 denote instantaneous time unit and the coefficient curve, respectively. The measurement error (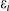) is assumed to be distributed normally with mean 0.

#### Non- Parametric Analysis of the Birth Outcomes

Indicator variables for the season during each given gestational month were used as a proxy of maternal exposure to PAHs. Our prior observations demonstrated that the season of PAH monitoring is a precise and accurate indicator of personal exposure (R^2^ = 0.74; all regression coefficients >0.96) [25]. The calendar months between conception and delivery dates were calculated based on last menstrual date- or sonogram-based gestational age [35]. To preclude multicollinearity in the birth outcome models, the indicator variables were calculated for each newborn's first, third and the last gestational month. The season during given gestational month was coded as winter if it corresponded to the months December–February. The remaining calendar months were set as the reference. 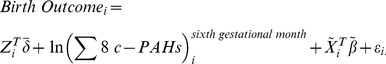
(4)Here, *Z* represents a vector of the winter indicator variable during the first, third, and the ninth gestational months, and 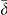 represents the respective regression coefficients. Since personal air monitoring was conducted in all women in the cohort during sixth gestational month, we accounted for this with the term, 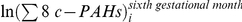.

#### Cross-Validation of the predicted PAH exposure

The prediction ability of our final model for a future observation is evaluated using “leaving-one-out” strategy of cross validation. Specifically, we fit the model with one observation deleted at each time and use that observation as the test sample to calculate the prediction error, defined as the difference square between observed and predicted. We do this for each observation and average the prediction error over. That is, we calculate
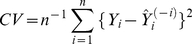
(5)where 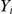 denote the observed outcome of the i^th^ observation and 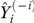 denote the predicted outcome of that observation with regression applied to the data with 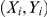 deleted. The statistical analysis was conducted in R, version 2.5.1 [37], SAS version 9.1.3 (SAS Institute Inc., Cary, NC, USA), and PASW Statistics version 14.0 (SPSS Inc., 2009, Chicago, IL, USA). Figures were generated in R 2.5.1 and PASW Statistics version 14.0.

## Results

The demographic characteristics of the mother-newborn pairs are shown in Table 1. The majority of the pregnant women did not have any risk factors for adverse birth outcomes (e.g., secondhand cigarette smoke, low educational attainment, or absence of spouse during the present pregnancy) or other sources of PAH exposure (i.e. routine consumption of grilled/barbecued food).

**Table 1:** Demographic and exposure characteristics of mother-newborn pairs.

Characteristics	*N (%)*	*Mean ± S.D.*
Mother's characteristics		
Age [years]	344 (100%)	28±4
Annual household income^a^		
Low <37,024 PLN	232 (67.4%)	
Medium 37,024–74,048 PLN	16 (4.7%)	
High >74,048 PLN	1 (0.3%)	
Refused/Don't know	95 (27.6%)	
Education		
<high school	37 (10.8%)	
high school graduate	91 (26.5%)	
Attained>high school	216 (62.8%)	
Parity [yes]	126 (36.6%)	
Currently married [yes]	320 (93.0%)	
Routinely consumed high level of PAH-laden food items^b^	49 (14.2%)	
Pregnant women with daily alcohol intake^c^	4 (1.2%)	
Season of delivery		
Winter (Dec–Feb)	85 (24.7%)	
Spring (March–May)	94 (27.3%)	
Summer (June–Aug)	90 (26.2%)	
Fall (Sep–Nov)	75 (21.8%)	
Secondhand cigarette smoke		
Non-smoker home	286 (83.1%)	
Exposed to ≤4 hrs/day	46 (13.4%)	
Exposed to 5+ hrs/day	12 (3.5%)	
Newborn characteristics		
Gender [female]	175 (50.9%)	
Newborn cotinine (*n*g/ml)	228 (66.3%)	0.319±0.882
Birthweight (g)		3430±491
Birth length (cm)		55±3
Birth head circumference (cm)		33.93±1.48

a For 2003, 1 US dollar was equal to 4.07 Poland Zlotych (PLN).

b Reported taking at least one smoked, grilled or barbequed food >twice/wk.

c Drank at least one glass of wine, beer, or liquor per day during pregnancy.

The prevalence of preterm delivery (4.7%, n = 16) and low birthweight (0.6%, n = 2) was low. Overall, mean FGR (mean ± S.D., 104±12%, range, 70–149%) and mean Ponderal Index (mean ± S.D., 2.11±0.21, range, 1.60–3.12) suggest that the birthweight distribution of the present cohort were comparable to the Polish population means. In this cohort, FGR demonstrates a high internal validity as a summary index of IUGR, and is consistent with trends in birthweight, birth length, birth head circumference, and Cephalization Index (Table 2). The severity of FGR was inversely correlated with the Cephalization Index (Pearson's coefficient = −0.68, *p-value<0.001*). At the same time, severe FGR newborns (<75%) were not significantly thinner (i.e. lower Ponderal Index) than non-FGR newborns (Table 2).

**Table 2:** Correlation between fetal growth ratio with other anthropometric indicators^a^.

fetal growth ratio (%)	N	Birthweight [g]	Birth Length [cm]	Birth Head Circumference [cm]	Ponderal Index [g/cm^3^×100]	Cephalization Index [µm/g]
		*Mean ± SD*	*Mean ± SD*	*Mean ± SD*	*Mean ± SD*	*Mean ± SD*
		*(min – max)*	*(min – max)*	*(min – max)*	*(min – max)*	*(min – max)*
Non-case	326	3482±436	55±3	34.07±1.34	2.12±0.21	99±11
(fetal growth ratio ≥85%)		(2150–4700)	(46–64)	(31.00–39.00)	(1.60–3.12)	(74–153)
Mild	11	2657±231	51±2	31.91±0.70	1.96±0.20	121±12
(80–84.99%)		(2210–2850)	(50–55)	(31.00–33.00)	(1.68–2.27)	(109–145)
Moderate	2	2225±460	48±3	31.50±0.71	2.00±0.06	144±27
(75–79.99%)		(1900–2550)	(46–50)	(31.00–32.00)	(1.95–2.04)	(125–163)
Severe	3	2190±295**	48±3**	30.67±0.58**	2.03±0.20	142±21**
(<75%)		(1870–2450)	(44–50)	(30.00–31.00)	(1.80–2.20)	(127–166)
Overall mean^b^	342	3436±477	55±3	33.96±1.42	2.11±0.21	100±13
		(1870–4700)	(44–64)	(30.00–39.00)	(1.60–3.12)	(75–166)

a Severity was defined as non-case (≥85%), mild (80–84.99%), moderate (75–79.99%), and severe (<75%) [46].

b Sample size is reduced from 344 to 342 because two newborns had gestational age of 43 and 29 weeks, which fell outside the plausible range [34].

** Test of linear trend with increasing severity of FGR, p<0.001.

### I. Estimated Personal Chronic PAH Exposure

**Figure 1:**
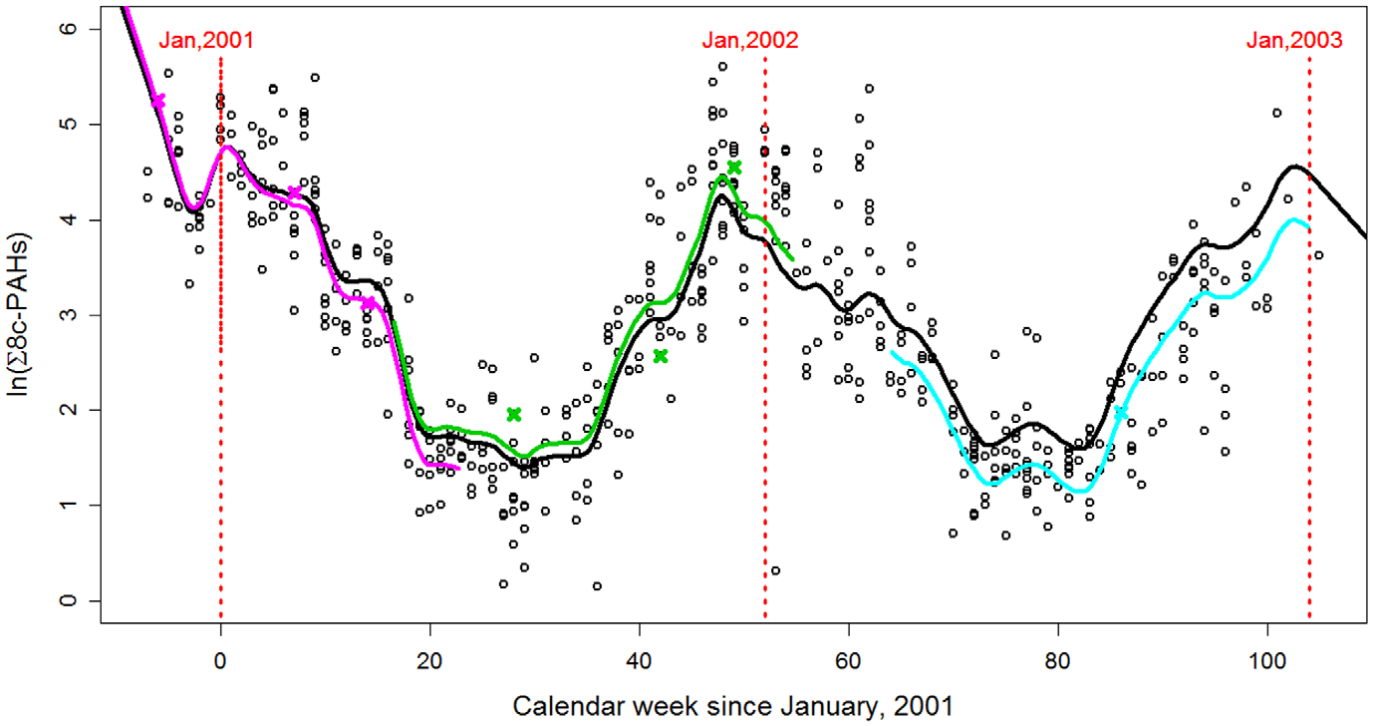
The observed and predicted individual gestational Σ8 c-PAH exposure using semi-parametric mixed effects model^a^. a. Black, open circle represents the observed concentration of Σ8 c-PAHs. Black line represents the pooled cohort mean during the entire monitoring period. Three persons were randomly selected to demonstrate estimated personal Σ8 c-PAHs exposure during her entire pregnancy period (in color). Based on the semi-parametric mixed effects model, Pearson's correlation coefficients between observed vs. predicted prenatal exposure were 0.91, 0.98 and 0.96 for the third, sixth and the eighth month.

Consistent with our earlier analysis [25], the three illustrative subjects shown in Figure 1 demonstrate that their chronic individual-level of exposure is predominantly influenced by ambient PAHs. The same subjects show that within-person variability in the estimated PAH exposure across the seasons was markedly larger than the between-person variability in the estimated PAH exposure during a same season (Figure 1). Prediction error between the predicted vs. observed exposure to Σ8 c-PAHs was 0.43 for the 344 newborns (see Figure S1).

### II. Mean PAH Effect Sizes by Trimester on Symmetric Fetal Growth Restriction

#### a. Random Effects Model-Estimated PAH Exposure and Mean Effect By Trimester

**Figure 2:**
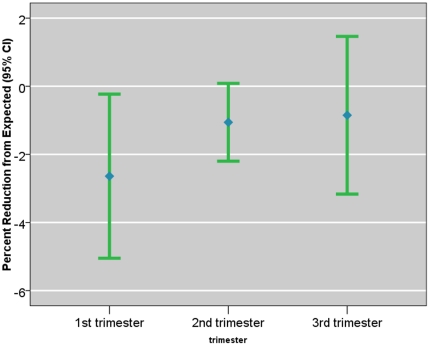
Mixed Effects Model Estimated ln-unit Σ8 C-PAH Exposure and Their Effects on Fetal Growth Ratio. The ln Σ8 c-PAHs effects on FGR was estimated as a mean effect per trimester-wise exposure and 95% confidence interval.

Using random effects model, one ln-unit exposure to Σ8 c-PAHs during the first trimester was associated with the largest mean reduction in FGR (−3%, 95% CI, −5 to −0%), compared to the mean reduction during the second or the third trimester (see Figure 2).

In addition, the same unit increase in exposure was associated with the largest mean reduction in birthweight during the first trimester (−105 g, 95% CI, −188 to −22 g), compared to the second (−36 g, 95% CI, −76 to 4 g), or the third trimester (−44 g, 95% CI, −123 to 36 g). For the birth length, a one ln-unit increase in exposure during the first trimester was associated with the largest mean reduction (−0.78 cm, 95% CI, −1.30 to −0.26 cm) compared to the same unit of exposure during the second trimester (−0.24 cm, 95% CI, −0.49 to 0.01 cm) or during the third trimester (−0.57 cm, 95% CI, −1.07 to −0.07 cm). However, one ln-unit of exposure was associated with a similar reduction in birth head circumference during the first (−0.11 cm, 95% CI, −0.39 to 0.17 cm) and the second trimesters (−0.13 cm, 95% CI, −0.26 to 0.00 cm). All models controlled for the mother's pre-pregnancy body mass index, gestational age, gender and parity.

#### b. Semi-Parametric Mixed Effects Model-Based Exposure Estimation

**Figure 3:**
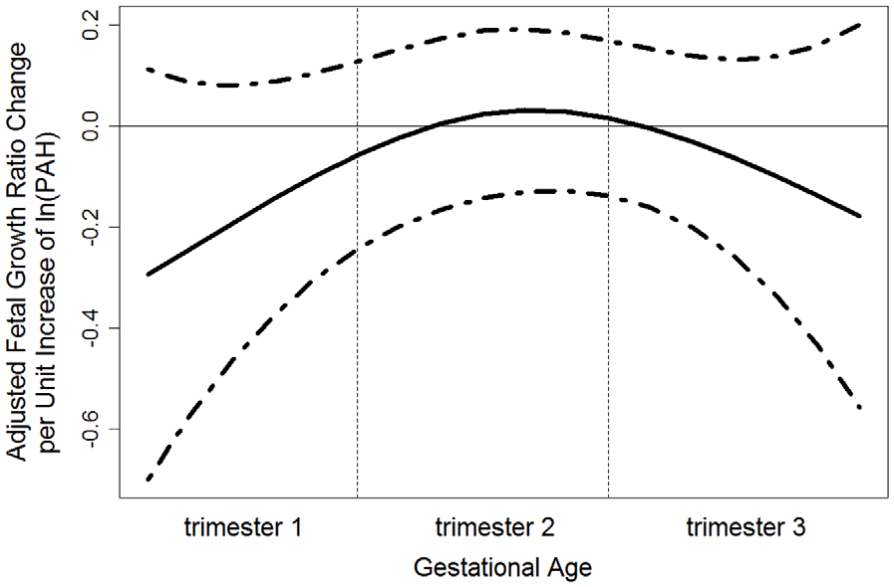
Semi-Parametric Mixed Model Estimated ln-Unit Σ8 C-PAH Exposure and Their Point-Wise Effects on Fetal Growth Ratio. Exposure was estimated using semi-parametric mixed effects model. Figure shows continuous, point-wise effect throughout the trimester based on functional linear model. The bold line shows regression coefficient per natural-log (ln) unit exposure to airborne PAHs. The dotted lines show point-wise 95% confidence interval.

Figure 3 shows the point-wise PAH effect on FGR throughout the gestational period. Within this cohort, the newborns that experienced their highest exposure during the earliest gestational weeks were associated with the largest point-wise FGR decrement, compared to other newborns that experienced lower exposure during the same period. Among the full-term newborns, a high ambient PAH concentration during the earliest gestational weeks is positively correlated with an elevated exposure during the last gestational months due to seasonal trend. Thus, a unit of PAH exposure during the first four (or, the last four) gestational weeks is associated with a largest reduction in FGR. Using the semi-parametric exposure estimation approach, a unit of PAH exposure during the first gestational month was associated with a mean reduction in FGR of 0.244% (95% CI, −0.574, 0.086%), compared to the effect size during the reference period (6^th^ month). Similarly, a unit PAH exposure during the 9^th^ gestational month was associated with a 0.178% (95% CI, −0.558, 0.201%) reduction in FGR after adjusting for the effects during other gestational months.

#### c. Non-parametric Comparison of ambient PAH Levels and Birth Outcomes

Compared to the newborns who experienced winter during their 6^th^ gestational month, those that experienced winter (December–February period) during their 1^st^ gestational month had a significantly larger reduction in birthweight (−191 g, 95% CI, −316 to −67 g), birth length (−1.14 cm, 95% CI, −1.93 to −0.35 cm) and FGR (−5%, 95% CI, 9 to −2%) respectively. The models accounted for the same set of potential confounders (Table 3).

**Table 3:** Non-parametric markers of ambient PAH concentration during various gestational periods and birth outcomes^a^.

Nonparametric markers of ambient PAH concentration during various gestational periods	*Birthweight*	*Birth Length*	*Birth head circumference*	*Fetal Growth Ratio*	*Ponderal Index*	*Cephalization Index*
	*(n = 344)*	*(n = 344)*	*(n = 344)*	*(n = 340)*	*(n = 344)*	*(n = 344)*
	*R^2^ = 0.461*	*R^2^ = 0.348*	*R^2^ = 0.275*	*R^2^ = 0.093*	*R^2^ = 0.018*	*R^2^ = 0.505*
	[g] reduction from the mean (95% CI)	[cm] reduction from the mean (95% CI)	[cm] reduction from the mean (95% CI)	[%] reduction from the mean (95% CI)	[g/cm^3^×100] reduction from the mean (95% CI)	[µm/g] reduction from the mean (95% CI)
Season during 1^st^ month (winter vs. other)^b^	−191 (−316, −67)	−1.14 (−1.93, −0.35)	−0.38 (−0.80, 0.03)	−5.37 (−8.92, −1.82)	0.02 (−0.05, 0.08)	5 (1, 8)
Season during 3^rd^ month (winter vs. other)^b^	−122 (−226, −17)	−0.62 (−1.29, 0.05)	−0.37 (−0.72, −0.02)	−3.04 (−6.06, −0.03)	0.00 (−0.06, 0.06)	3 (0, 6)
Directly monitored personal c-PAH exposure, second trimester^c^	−67 (−110, −23)	−0.48 (−0.76, −0.20)	−0.20 (−0.34, −0.05)	−1.85 (−3.09, −0.60)	0.01 (−0.01, 0.04)	1 (0, 3)
Season during 9^th^ month (winter vs. other)^b^	−40 (−148, 68)	−0.36 (−1.05, 0.33)	−0.05 (−0.41, 0.32)	−0.69 (−3.78, 2.39)	0.02 (−0.04, 0.08)	1 (−2, 4)

a Model controls for gestational age (centered at mean and square term of the centered at mean), newborn gender, parity, and the mother's pre-pregnancy body mass index. C-section delivery is included only for the head circumference.

b To reduce multicollinearity in the models, the indicator variable was coded as the winter vs. other.

c Direct measurement of personal exposure was determined using a personal monitor for 48-hour period. The exposure variable was coded in natural-log scale.

### III. Prenatal PAH Exposure and Asymmetric Fetal Growth Restriction

**Figure 4:**
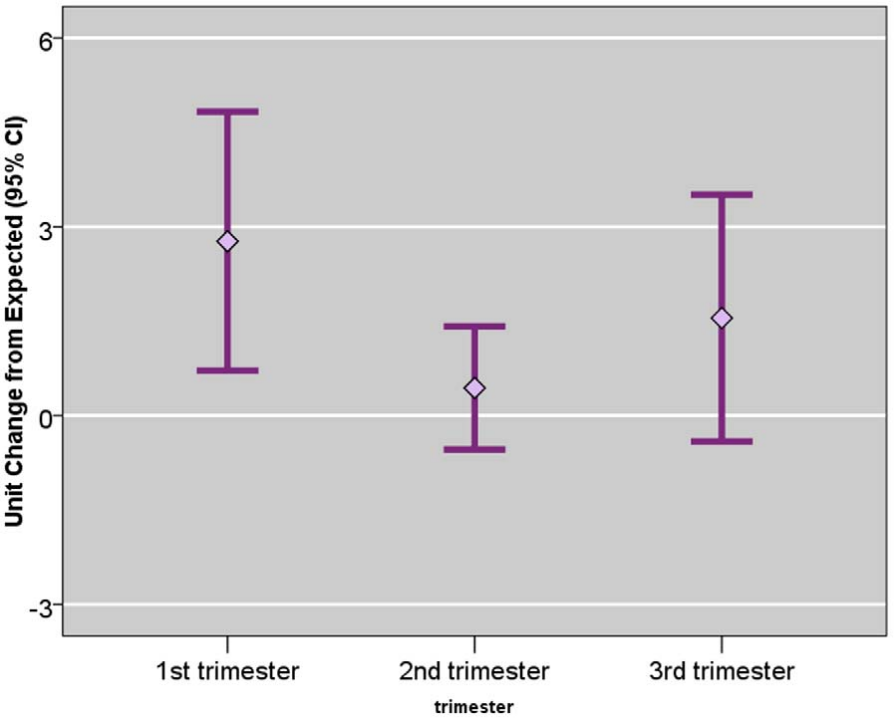
Mixed Effects Model Estimated ln-unit Σ8 C-PAH Exposure and Their Effects on Cephalization Index. The ln Σ8 c-PAHs effects on the outcome was estimated as a mean effect per trimester-wise exposure and 95% confidence interval.

**Figure 5:**
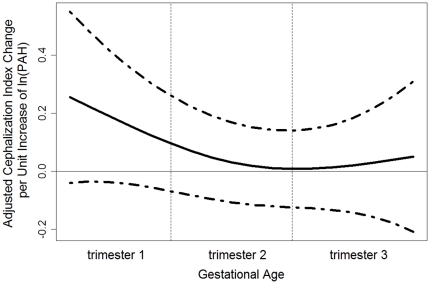
Semi-Parametric Mixed Model Estimated ln-Unit Σ8 C-PAH Exposure and Their Point-Wise Effects on Cephalization Index. Exposure was estimated using semi-parametric mixed effects model. Figure shows continuous, point-wise effect throughout the trimester based on functional linear model. The bold line shows regression coefficient per natural-log (ln) unit exposure to airborne PAHs. The dotted lines show point-wise 95% confidence interval.

**Figure 6:**
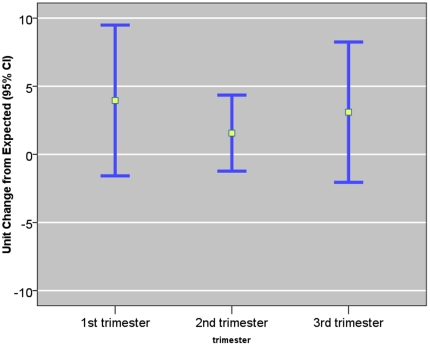
Mixed Effects Model Estimated ln-unit Σ8 C-PAH Exposure and Their Effects on Ponderal Index. The ln Σ8 c-PAHs effects on the outcome was estimated as a mean effect per trimester-wise exposure and 95% confidence interval.

One ln-unit ∑8 PAHs during the first trimester (using random effects model for prediction of chronic exposure) was associated with the largest mean elevation in Cephalization Index (3 μm/g, 95% CI, 1 to 5 μm/g), adjusting for the same set of confounders (Figure 4). Based on the semi-parametric exposure estimation method, the first month's exposure was associated with a highest point-wise elevation in Cephalization Index (0.22 μm/g, 95% CI, −0.04, 0.48 μm/g), compared to the ratio during the 6^th^ month (0.01 μm/g, 95% CI, −0.12, 0.14 μm/g, Figure 5). As the exposure during the earliest and the last gestational months are correlated, the same unit exposure was also associated with an elevation in Cephalization Index (e.g., 0.05 μm/g) during the 9^th^ month. Alternatively, based on consideration of non-parametric indicators, the newborns who experienced winter (December–February) during their 1^st^ gestational month, on average, had the largest elevation in Cephalization Index (5 μm/g, 95% CI, 1 to 8 μm/g), compared to the newborns who experienced winter during their 6^th^ gestational month (Table 3). On the other hand, prenatal PAH exposure was not associated with a significant reduction in Ponderal Index during any gestational period (Figure 6).

## Discussion

The identification of a “window of critical vulnerability” to ubiquitous air pollutants such as PAHs is a particularly important, yet challenging, question. Critical hurdle with answering this question regards the dose-response relationship of the xenotoxicant during a given age, which is inherently related to the host's susceptibility as well as the host's adaptiveness. Such exquisite sensitivity of the fetus and newborn to xenotoxicants is thought to be related to the immaturity of the developing immune systems; the rapid development of fetal organs; epigenetic mediation; and the fact that exposure per body weight is much higher than for adult exposure [38], [39], [40]. Thus, the age-specific measurement of PAH exposure is critical for the clarification of the severity and the type of IUGR [10], [22].

The present analysis supports the hypothesis that PAH exposure during the first trimester imparts the largest reduction in the markers of symmetric fetal growth restriction. Furthermore, we originally posited that elevated Cephalization Index represents a marker of asymmetric growth. However, our results suggest that it is, in fact, correlated with a symmetric fetal growth restriction. Prenatal PAH exposure during the earliest gestational months was associated with a consistent effect on Cephalization Index and FGR. Also, we saw no evidence that PAH exposure increases the likelihood of acute fetal growth restriction, as indicated by the Ponderal Index.

The strong inverse correlation between FGR and Cephalization Index in our results is also supported by similar results in the murine model [16]. B[*a*]P administration during organogenesis interfered with preliminary synapse formation during the earliest weeks of gestation and induced the largest disproportionate increase in Cephalization Index [16]. Furthermore, gestational B[*a*]P exposure postnatally inhibited the cortical region for learning and memory [16].

Our present observation of the largest unit effect during the first trimester suggests that PAHs might influence the rate of fetal growth. Other epidemiologic investigations independently observed that fetal growth rate is programmed during the earliest gestational period, resulting in a progressively larger deficit as gestation matures [18], [19], [20].

An adverse intrauterine environment, particularly during the early pregnancy period, is hypothesized to switch on the survival mechanism of the fetus by protecting vital organs such as brain and heart while suppressing the development of other systems [41]. Recent clinical examinations in various populations have demonstrated that a significantly slower growth rate begins in the earliest gestational weeks for growth restricted newborns [18], [19], [20]. Among a group of singleton newborns who were longitudinally followed from the 12^th^ week of gestation to delivery by ultrasound, a significant difference in inter-individual rate of fetal growth was evident in terms of fetal abdominal and head circumference, as well as femur diaphysis length [20]. Starting around the 13^th^ week, significantly slower fetal growth velocity was evident for those who were born in the lowest third percentile of birth weight. This difference in fetal growth velocity remained constant throughout gestation after maternal anthropomorphic characteristics (including pre-pregnancy weight, height and weight gain) were accounted for [20].

In some populations, fetuses that are born small-for-gestational age or with low birthweight, are at greater risk of neurodevelopmental delays [28], impaired lung function [42], asthma symptoms [43] throughout childhood, as well as cardiopulmonary diseases [41] during adulthood, including hypertension, and atherosclerosis, as well as diabetes [44]. Our personal, indoor, and outdoor air monitoring of 344 women represents one of the largest and most comprehensive PAH exposure assessment campaigns to date. The airborne PAH exposure in Krakow, Poland, represents a typical exposure scenario in countries dependent on coal-burning for heat and power generation [24]. Ambient PAH concentrations differ by more than two orders of magnitude between summer and winter with overall spatial homogeneity in concentration during given season [24]. The extreme seasonal fluctuation of ambient PAH levels was instrumental in obtaining valid and precise predicted personal PAH exposure concentrations during the unmonitored months. Spearman's coefficients for concurrent personal, indoor and outdoor measurements ranged between 0.96–0.98 [25]. Thus, the spatial variability in personal, indoor and outdoor PAH concentration (within a given household) was very small during a typical air pollution episode (Figure 1). For example, the maximum values for personal B[*a*]P exposure concentration (42.23 ng/m^3^) in our cohort and ambient B[*a*]P level (200 ng/m^3^), as reported by Junninen et al. (2009), represent two of the highest values of the compound that have ever been documented. Accordingly, the cohort's mean exposure over the monitoring period was weighed heavily in estimating the personal exposure of other newborns during their unmonitored months. Pearson's correlation coefficients between observed vs. predicted personal exposure concentration were 0.91, 0.98, and 0.96 at the 3^rd^, 6^th^, and 8^th^ gestational month, respectively.

Another key strength of the study is the application of stringent enrollment criteria. Only young Polish women (18–35 years of age), non-smoking, healthy (i.e., free of diabetes, hypertension, or known HIV, nonusers of other tobacco products or illicit drugs) were targeted. In addition, only those who received adequate prenatal care (i.e. enrolled in the clinic between 8^th^ to 13^th^ week of pregnancy) were included. Thus, several important confounders have been precluded. At the same time, several study limitations warrant consideration. As our strict enrollment criteria precluded notable sources of confounding, our mothers and newborns cohort is not representative of the general population. Furthermore, only those fetuses who survived beyond the 8–13^th^ gestational weeks were recruited into the study. Thus, PAH effects in the general population might be different than in the present cohort.

It is currently unknown whether other constituents of coal combustion by-products, including Cadmium (Cd), Nickel (Ni), Arsenic (As), and Lead (Pb) affect birth outcomes through a mechanism common with PAHs. Thus, our results cannot rule out confounding effects from other airborne correlates of PAHs, particularly during the winter. Another limitation of the study is the fact that we did not consider the genetic polymorphisms of the developing fetus and/or the mothers for the risk of IUGR. In our earlier analyses, both maternal and newborn haplotypes of the cytochrome 450 genes CYP1A1 significantly augmented PAH effects on children's neurocognitive development once they reached the age of 1, 2, and 3 respectively [45]. It is plausible that the genes involved in PAH metabolic activation or detoxification may also modify PAH exposure risks on IUGR.

Cyclic fluctuation in ambient PAH concentration influenced the mean personal exposure during the first gestational month so that it was positively correlated with exposure during the ninth gestational month. While we statistically adjusted for this correlation in exposure, future research should quantify the fetal growth rate in real-time during gestation, rather than determine the absolute size decrement at birth. Such a measurement would also be useful in early detection of IUGR cases.

### Conclusion

The identification of a “window of critical vulnerability” to ubiquitous air pollutants such as PAHs is a particularly important, yet challenging, question. The challenge surrounding this question stems, at least partly, from the fact that the dose-response relationship of the xenotoxicant during a given age is inherently related to the host's susceptibility as well as the host's adaptiveness. Furthermore, exposure duration is chronic, yet variable. Our results based on several alternate exposure estimation approaches suggest that one ln-unit PAH exposure during the first trimester, and the first gestational month in particular, increases the risk of FGR reduction and Cephalization Index elevation, respectively. On the other hand, no gestational period was associated with a marked reduction in Ponderal index. Reduction in birthweight, birth length and FGR, as well as an elevated CI predict mortality and morbidity risks in newborns and compromised cognitive development in children. In addition, ambient PAH concentrations in Krakow are typical of regions dependent on coal-burning for heat and power generation [24]. The present data support the need for a multinational coal-combustion abatement strategy for the protection of pregnant women and the embryo/fetus, particularly during the earliest stage of pregnancy.

## Supporting Information

Figure S1 **Model Fitting for the Semi-Parametric Mixed Model (** ***number of measurements = 489*** **).**
(TIF)

Table S1 **Frequency and Timing of Personal Air Monitoring.**
(DOCX)
