# Supplementary figures and images for: Fetal Window of Vulnerability to Airborne Polycyclic Aromatic Hydrocarbons on Proportional Intrauterine Growth Restriction
Source: PLoS One. 2012 Apr 24;7(4):e35464. doi: 10.1371/journal.pone.0035464 (PMC3335852; doi:10.1371/journal.pone.0035464)

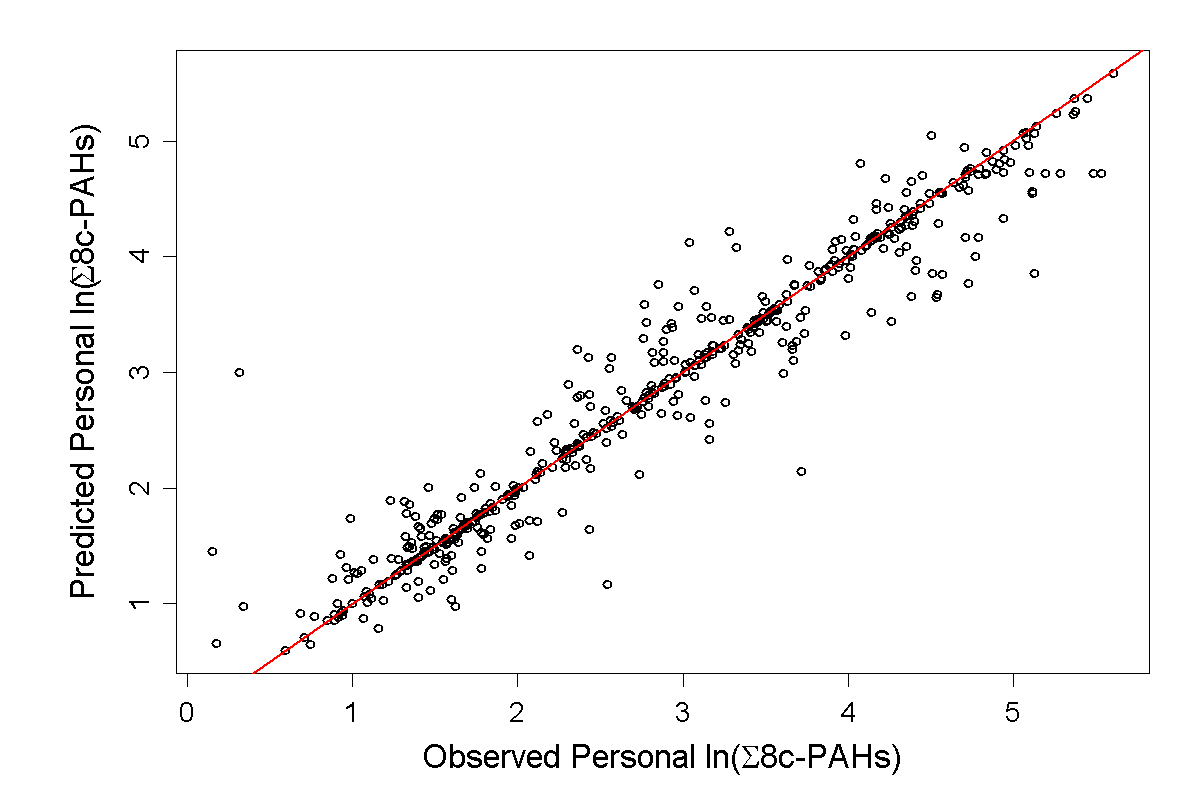

Supplement: Figure S1 — Model Fitting for the Semi-Parametric Mixed Model ( number of measurements = 489 ). (TIF) [file pone.0035464.s001.tif]
