# Supplementary material for: Fetal Window of Vulnerability to Airborne Polycyclic Aromatic Hydrocarbons on Proportional Intrauterine Growth Restriction
Source: PLoS One. 2012 Apr 24;7(4):e35464. doi: 10.1371/journal.pone.0035464 (PMC3335852; doi:10.1371/journal.pone.0035464)
Supplement: Table S1 — Frequency and Timing of Personal Air Monitoring. (DOCX) [file pone.0035464.s002.docx]

Table S1: Frequency and Timing of Personal Air Monitoring.

|  | n | first trimester  (no. monitoring) | second trimester  (no. of monitoring) | third trimester  (no. of monitoring) | total  monitoring  per individual |
| --- | --- | --- | --- | --- | --- |
| Singly  monitored  individuals | 208 |  | 208 |  | 208 |
|  | 59 |  |  | 59 | 59 |
|  |  |  |  |  |  |
| Repeatedly  monitored  individuals | 68 | 68 | 68 | 68 | 204 |
|  | 2 | 2 | 2 |  | 4 |
|  | 3 |  | 3 | 3 | 6 |
|  | 4 | 4 |  | 4 | 8 |
| time-point total | 344 | 74 | 340 | 75 | 489 |
